# The frailty index outperforms DNA methylation age and its derivatives as an indicator of biological age

**DOI:** 10.1007/s11357-017-9960-3

**Published:** 2017-01-14

**Authors:** Sangkyu Kim, Leann Myers, Jennifer Wyckoff, Katie E. Cherry, S. Michal Jazwinski

**Affiliations:** 1 Tulane Center for Aging and Department of Medicine, Tulane University Health Sciences Center, Box 8513, 1430 Tulane Ave., New Orleans, 70112 LA USA; 2 Department of Global Biostatistics and Data Science, School of Public Health and Tropical Medicine, Tulane University Health Sciences Center, New Orleans, LA USA; 3 Department of Psychology, Louisiana State University, Baton Rouge, LA USA

**Keywords:** Aging, Biological age, Frailty, DNA methylation, Mortality

## Abstract

The measurement of biological age as opposed to chronological age is important to allow the study of factors that are responsible for the heterogeneity in the decline in health and function ability among individuals during aging. Various measures of biological aging have been proposed. Frailty indices based on health deficits in diverse body systems have been well studied, and we have documented the use of a frailty index (FI_34_) composed of 34 health items, for measuring biological age. A different approach is based on leukocyte DNA methylation. It has been termed DNA methylation age, and derivatives of this metric called age acceleration difference and age acceleration residual have also been employed. Any useful measure of biological age must predict survival better than chronological age does. Meta-analyses indicate that age acceleration difference and age acceleration residual are significant predictors of mortality, qualifying them as indicators of biological age. In this article, we compared the measures based on DNA methylation with FI_34_. Using a well-studied cohort, we assessed the efficiency of these measures side by side in predicting mortality. In the presence of chronological age as a covariate, FI_34_ was a significant predictor of mortality, whereas none of the DNA methylation age-based metrics were. The outperformance of FI_34_ over DNA methylation age measures was apparent when FI_34_ and each of the DNA methylation age measures were used together as explanatory variables, along with chronological age: FI_34_ remained significant but the DNA methylation measures did not. These results indicate that FI_34_ is a robust predictor of biological age, while these DNA methylation measures are largely a statistical reflection of the passage of chronological time.

## Introduction

Degenerative biological changes and functional decline occur with advancing age, increasing the incidence of disorders, diseases, and mortality. Thus, biological aging proceeds in tandem with chronological age. However, the pace and extent of age changes vary among individuals at any given chronological age (Mitnitski et al. 2002a; Karasik et al. 2005). Thus, the term biological aging has been developed to conceptualize the fact that individuals differ in their manifestation of age changes as they age chronologically. The heterogeneity in biological aging among chronological age peers necessitates a reliable measure of biological or functional age separate from chronological age.

A good measure of biological age should reflect age-related changes occurring at various biological levels. One of the best characterized measures of biological age is the frailty (or deficit) index (Rockwood et al. 1994, 1999; Fried et al. 2001; Mitnitski et al. 2001; Kulminski et al. 2007a, b). This composite index is expressed as the proportion of health deficits accumulated by individuals among a set of health items surveyed throughout the body, and it can be calculated at any given chronological age (Mitnitski et al. 2001). The health items (variables) include various signs, symptoms, laboratory measurements, disabilities, and diseases. A frailty index calculated from about 20 to 100 health variables gives reliable and comparable results (Mitnitski et al. 2006; Rockwood et al. 2007; Rockwood and Mitnitski 2007; Searle et al. 2008). Frailty indexes have been extensively examined and used in various studies (Mitnitski et al. 2001, 2002a, b; Kulminski et al. 2007a, b; Matteini et al. 2010).

We developed a frailty index called FI_34_ (Kim et al. 2013; Kim and Jazwinski 2015). Composed of 34 common health and function variables, FI_34_ increases exponentially with age, indicating declining health and function ability. The rate of increase accelerates approximately 2~3 % annually, and the rate of increase and the pattern of aging displayed by the hierarchical clustering of the component variables differ among offspring of long-lived versus short-lived parents (Kim et al. 2013), which indicates a genetic basis of the frailty index. Indeed, an estimate of the heritability (narrow sense) of FI_34_ is relatively high (0.39). A survival analysis indicates that FI_34_ predicts mortality better than does chronological age (Kim et al. 2013). This is a critical determination. Frailty and deficit indices, such as FI_34_, increase with chronological age. Thus, they could simply be a surrogate for the passage of calendar time. The fact that FI_34_ predicts mortality/survival better than does the simple passage of time indicates that it is more than a naive chronometer. Rather, it is a metric of biological age, a complex and intrinsic feature of an organism.

Physiologic factors associated with FI_34_ have been identified. Elevated levels of resting metabolic rate are linked to higher FI_34_ scores in nonagenarians, indicating an increased energy demand for basic body functioning with declining health (Kim et al. 2014). This association of energy metabolism with healthy aging of the oldest old has various underlying factors that operate in a gender-specific manner (Kim et al. 2016a, b). In female nonagenarians, fat mass and fat-free mass are important contributors to healthy aging; in male nonagenarians, however, tissue quality rather than body composition is important. In addition, genetic factors that are associated with FI_34_ have been identified, which include *UCP2* and *UCP3*, in females, and *XRCC6*, and *LASS1*, in males. Also, non-coding genomic regions at 12q13-14 that appear to have regulatory function are related to healthy aging (Kim et al. 2015). Thus, FI_34_ has been useful in identifying both genetic and phenotypic factors related to healthy aging.

A different type of age measure was proposed based on leukocyte DNA methylation. DNA methylation levels at many CpG sites in the genome are correlated with chronological age (Fraga et al. 2005; Rakyan et al. 2010; Bocklandt et al. 2011; Koch and Wagner 2011; Lin et al. 2016), and subsets of such CpG sites have been used in epigenetic models of aging. Hannum et al. obtained a “predicted age” from DNA methylation levels at 71 CpG sites and calculated an “apparent methylomic aging rate (AMAR)” for each individual by dividing the predicted age by the chronological age (Hannum et al. 2013). AMAR greater than 1 was interpreted to mean “fast aging,” whereas AMAR less than 1 to mean “slow aging.” Similarly, Horvath selected 353 CpG sites in which DNA methylation levels are highly correlated with chronological age (Horvath 2013). He transformed subjects’ chronological ages (1 and 2 below), used a linear regression to describe the relationship between the transformed age and the DNA methylation levels of the 353 CpG sites that are highly correlated with chronological age “weighted” by their regression coefficients to maximize the overall relationship (3), and then took the “inverse” of this linear regression to calculate the DNAmAge of each subject (4):

1 $$ F\left(\mathrm{age}\right)= \log \left(\mathrm{age}+1\right)- \log \left(\mathrm{adult}\kern0.30em \mathrm{age}+1\right),\kern0.30em \mathrm{if}\kern0.30em \mathrm{age}\le \mathrm{adult}\kern0.30em \mathrm{age}\kern0.30em \left(=20\right) $$

2 $$ F\left(\mathrm{age}\right)=\left(\mathrm{age}-\mathrm{adult}\kern0.30em \mathrm{ag}\mathrm{e}\right)/\left(\mathrm{adult}\kern0.30em \mathrm{ag}\mathrm{e}+1\right),\kern0.30em \mathrm{i}\mathrm{f}\kern0.30em \mathrm{a}\mathrm{g}\mathrm{e}>\mathrm{adult}\kern0.30em \mathrm{ag}\mathrm{e}\kern0.30em \left(=20\right) $$

3 $$ F\left(\mathrm{age}\right)={b}_0+{b}_1{CpG}_1+{b}_2{CpG}_2\dots {b}_{353}{CpG}_{353}+\mathrm{error} $$

4 $$ \mathrm{DNAmAge}=\mathrm{the}\kern0.30em ``\mathrm{inverse}"\kern0.30em \mathrm{of}\kern0.30em \mathrm{the}\kern0.30em \mathrm{function}\kern0.30em \mathrm{F}\kern0.30em \mathrm{in}\kern0.30em (3) $$

Not surprisingly based on its dependence on chronological age, the DNAmAge is an excellent correlate of chronological age. Indeed, it is almost as accurate as the age on drivers’ licenses. Subsequently, the potential of DNAmAge-derived measures as indicators of biological age have been explored (Horvath et al. 2015; Marioni et al. 2015; Breitling et al. 2016; Chen et al. 2016; Christiansen et al. 2016; Perna et al. 2016). One of them, AgeAccelerationDiff (AgeDiff hereafter), is the difference between DNAmAge and chronological age. AgeDiff was associated with mortality (Marioni et al. 2015; Christiansen et al. 2016; Perna et al. 2016). It was also associated with a frailty index similar to our FI_34_ but having a much greater emphasis on activities of daily living and a substantially lesser focus on cognitive function (Breitling et al. 2016). Likewise, the residual of the linear regression of DNAmAge on chronological age, AgeAccelerationResidual (AgeResid hereafter), was associated with longevity and mortality (Horvath et al. 2015; Chen et al. 2016). These findings indicate that these DNAmAge-derived measures may represent biological age.

In this article, we compared DNAmAge measures with FI_34_ side by side and assessed the effectiveness of each of these measures in predicting mortality. Our results indicate that FI_34_ uniformly outperforms the DNAmAge measures.

## Materials and methods

### Subjects and health data

Subjects in this study are 262 unrelated individuals randomly selected from the Louisiana Healthy Aging Study cohort (Table 1). Age was based on documentary evidence supported by demographic questionnaires. Only Caucasians, inferred genetically, were included in data analyses to avoid population confounding (Jazwinski et al. 2010). Details of data collection and calculation and characterization of FI_34_ were described elsewhere (Kim et al. 2013). All participants provided informed consent according to the protocol approved by the respective Institutional Review Boards.

**Table 1:** Summary statistics of the study sample (*N* = 262; 206 deceased)

Measure	Group	Range	Mean ± SD	Male vs. female^a^
Age	All (262)	60∼103	86 ± 10	*P* = 0.28
	Male (103)	60∼99	85 ± 11	
	Female (159)	60∼103	87 ± 9	
FI_34_	All	0.0097∼0.49	0.22 ± 0.092	*P* = 5.2e-5
	Male	0.0097∼0.45	0.19 ± 0.085	
	Female	0.032∼0.49	0.23 ± 0.091	
DNAmAge	All	32∼110	78 ± 12	*P* = 0.45
	Male	43∼110	78 ± 13	
	Female	32∼107	78 ± 12	
AgeDiff	All	−41∼29	−8 ± 10	*P* = 0.0025
	Male	−41∼19	−6 ± 10	
	Female	−41∼29	−9 ± 10	
AgeResid	All	−36∼36	0 ± 10	*P* = 0.0086
	Male	−31∼28	1.8 ± 10	
	Female	−36∼36	−1.2 ± 9	

*SD* standard deviation

^a^Wilcoxon rank sum tests between the two gender groups

### DNA methylation data and analysis

Genomic DNA was isolated from blood specimens, and 500 ng of each genomic DNA sample was treated with bisulfite using the EZ-96 DNA methylation Kit (Zymo Research). DNA methylation data were obtained using the Infinium HumanMethylation450K BeadChip Kit (Illumina) at the University of Utah Genomics Core Facility. Data preprocessing and quality control were done using the R package RnBeads (Assenov et al. 2014). DNA methylation probes with the detection *P* value greater 0.05 were excluded from beta value calculation. DNA methylation age (“DNAmAge”) of each subject was obtained using the Online Age Calculator (https://dnamage.genetics.ucla.edu/) (Horvath 2013), with the recommended default setting. The DNAmAge was calculated directly without retraining the model on the DNA methylation data from our study population, as the online calculator was developed using a large collection of genome-wide methylation datasets. The output from the online calculator also contains cell count estimates of various leukocyte types, which were used as additional covariates in Cox proportional hazard regression analysis. All the statistical analyses were performed using R (R_Core_Team 2016).

## Results

### Characteristics of the study cohort

Our study sample consists of 262 Caucasians whose ages range from 60 to 103 (Table 1). Of these, 206 (79 %) were deceased at the time of follow-up just prior to this analysis, and the average elapsed time-to-death from entry into the study was 4.4 years. Females and males did not differ in mean chronological ages and mean DNAmAges, but the two gender groups significantly differed in FI_34_, AgeDiff, and AgeResid. FI_34_ was higher in females (*P* = 5.2e-5), as described previously (Kim and Jazwinski 2015). On the other hand, AgeDiff and AgeResid were higher in males (*P* = 0.0025 and 0.0086, respectively), which concurs with the higher AMAR in males (Hannum et al. 2013).

### DNAmAge and FI_34_ are correlated with age

**Fig. 1:**
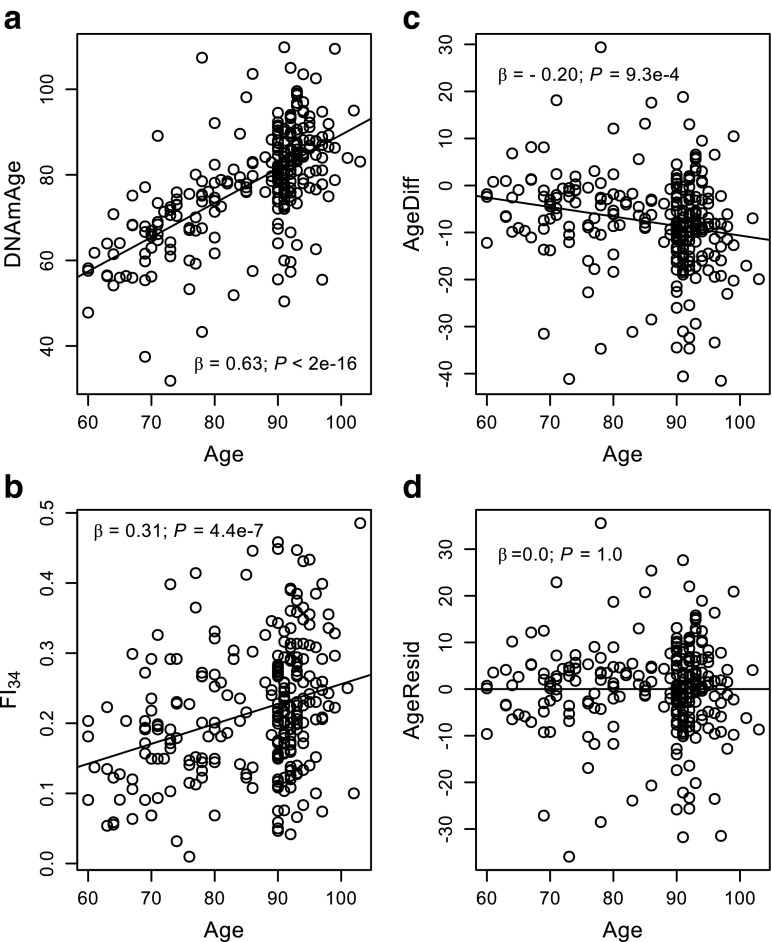
Scatter plots of chronological age (Age) by DNAmAge (**a**), FI_34_ (**b**), AgeDiff (**c**), and AgeResid (**d**). Each regression line is from the corresponding standardized simple linear regression, whose *β* value equals the correlation coefficient *r*

DNAmAge is derived from transformed chronological age in a statistical model in which the chronological age is regressed on the methylation status of 353 CpG sites throughout the genome (Horvath 2013). Thus, DNAmAge is expected to be highly correlated with chronological age. For example, the average correlation coefficients were 0.97 and 0.96 for all training and test data sets, respectively (Horvath 2013). Likewise, in our sample, DNAmAge was significantly correlated with chronological age (*r* = 0.63, *P* < 2.0e-16; Fig. 1a). FI_34_ was also correlated linearly with chronological age as health and function deficits tend to increase with age (*r* = 0.31, *P* = 4.4e-7; Fig. 1b), although an exponential model describes the relationship better (Kim et al. 2013; Kim and Jazwinski 2015). AgeDiff was also correlated with chronological age (*r* = −0.20, *P* = 9.3e-4; Fig. 1c), while AgeResid was not (Fig. 1d).

**Fig. 2:**
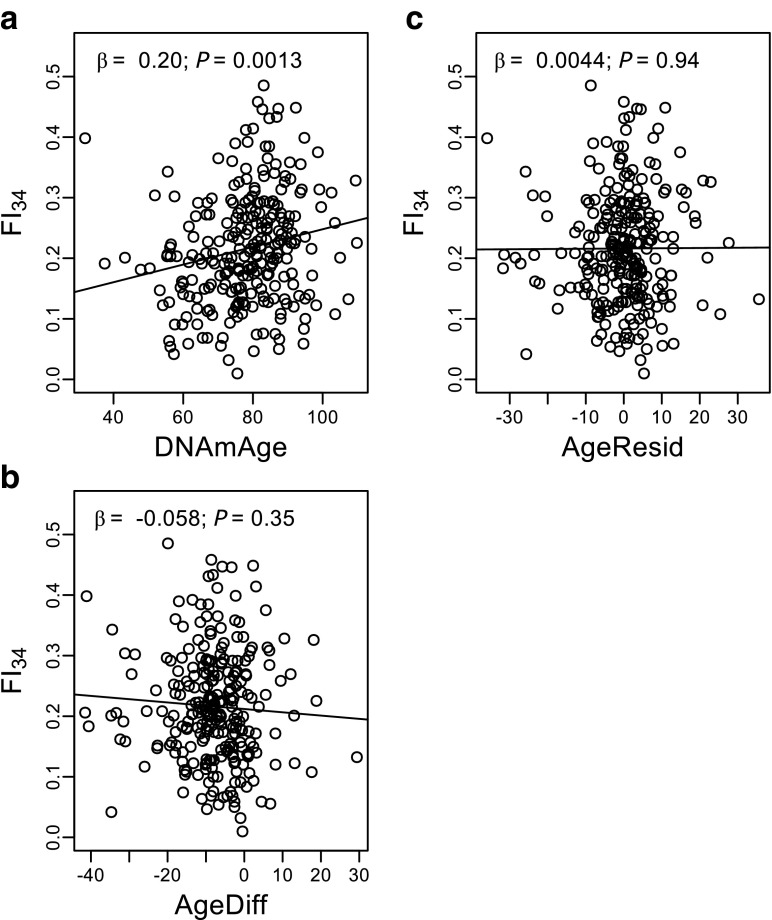
Scatter plots of FI_34_ by DNAmAge (**a**), AgeDiff (**b**), and AgeResid (**c**). Each regression line is from the corresponding standardized simple linear regression, whose *β* value equals the correlation coefficient *r*

As both FI_34_ and DNAmAge were correlated with chronological age, FI_34_ and DNAmAge were correlated with each other (*r* = 0.20, *P* = 0.0013; Fig. 2a). However, the correlation disappeared when adjusted for chronological age (*P* > 0.5 by partial correlation), indicating that without the age dependence, DNAmAge, and FI_34_ are unrelated. AgeDiff and AgeResid were not correlated with FI_34_ (Fig. 2b, c).

### DNAmAge is not a significant predictor of survival when adjusted for age

The performance of a measure of biological age is best assessed by its ability to predict mortality, which is the ultimate consequence of aging. For this purpose, most of the studies of DNAmAge measures used Cox proportional hazard regression. We applied the same statistical method to our survival data (Table 2). As expected, chronological age was a significant predictor of survival: The hazard of death was estimated to increase 13 % annually (*P* < 2.0e-16; model 1). DNAmAge increased the hazard of death by 5 % for a unit increase in DNAmAge (*P* = 3.3e-16; model 2). Likewise, FI_34_ was estimated to increase the mortality hazard by 5 % for an increase of FI_34_ by 0.01 (*P* = 4.8e-10; model 3). When present together as explanatory variables, DNAmAge and FI_34_ remained significant without affecting each other much (*P* = 2.6e-13 and 8.2e-7, respectively; model 4). In the presence of chronological age as an additional covariate, however, DNAmAge was no longer a significant mortality predictor (*P* = 0.63 and 0.61 in models 5 and 7, respectively), while FI_34_ still remained significant (*P* = 0.0054 and 0.0053 in models 6 and 7, respectively). These results confirm that DNAmAge and chronological age largely overlap with each other, but FI_34_ is a separate measure distinct from the two.

**Table 2:** Cox regression for time-to-death as a function of age, DNAmAge, or FI_34_ (*N* = 262)

Model	Variables	*b*	exp(*b*)	se(*b*)	*Z*	*P*	*R* ^2^	Wald test *P*
1	Age	0.12	1.13	0.011	11	<2.0e-16	0.48	<2.0e-16
2	DNAmAge	0.048	1.05	0.0059	8.2	3.3e-16	0.22	3.0e-15
3	FI_34_^a^	0.049	1.05	0.0079	6.2	4.8e-10	0.13	2.6e-09
4	DNAmAge	0.046	1.05	0.0063	7.3	2.6e-13	0.29	<2.0e-16
	FI_34_^a^	0.039	1.04	0.0079	4.9	8.2e-07		
5	Age	0.12	1.12	0.012	9.4	<2.0e-16	0.48	<2.0e-16
	DNAmAge	0.0037	1.00	0.0077	0.49	0.63		
6	Age	0.11	1.12	0.011	10	<2.0e-16	0.49	<2.0e-16
	FI_34_^a^	0.022	1.02	0.0080	2.8	0.0054		
7	Age	0.11	1.12	0.012	8.8	<2.0e-16	0.50	<2.0e-16
	DNAmAge	0.0050	1.00	0.0078	0.52	0.61		
	FI_34_^a^	0.022	1.02	0.0080	2.8	0.0053		
8	Age	0.11	1.12	0.013	8.6	<2.0e-16	0.51	<2.0e-16
	DNAmAge	0.0039	1.00	0.0084	0.47	0.64		
	FI_34_^a^	0.020	1.02	0.0085	2.4	0.016		
	WBC^b^	–	–		–	–		

All the regressions above contained sex as a covariate

*b* regression coefficient, *exp(b)* exponentiated *b*, *se(b)* standard error of *b*, *Z* the ratio of *b* to its standard error

^a^FI_34_ was multiplied by 100; therefore, the *b* value for FI_34_ is the hazard of death for an increase of FI_34_ by 0.01

^b^CD8.naive + CD8pCD28nCD45Ran + PlasmaBlast + CD4T + NK + Mono + Gran

Different types of leukocytes exist in blood, and their proportions may vary, depending on individuals’ health conditions at the time of blood collection. This leukocyte heterogeneity may confound estimation of intrinsic DNA methylation levels (Reinius et al. 2012). Methods to estimate leukocyte type counts were developed and have been often used to adjust DNA methylation measurements (Houseman et al. 2012, 2014; Accomando et al. 2014). Therefore, leukocyte type estimates were obtained using Horvath’s Online Age Calculator and included as additional covariates in our Cox regression analysis. The outcome of the leukocyte composition adjustment (model 8) was very similar to the outcome without the adjustment (model 7). The only noticeable difference is a slight decrease in the *Z* value of FI_34_ from 2.8 to 2.4, resulting in a higher *P* value (*P* from 0.0053 to 0.016). This small change is likely due to the redundancy of FI_34_ and leukocyte composition in reflecting health conditions.

The Cox regression analysis was repeated with AgeDiff (Table 3). A unit increase in AgeDiff was estimated to reduce the hazard by 2 % (*P* < 0.020; model 1). When present together, AgeDiff and FI_34_ remained significant without affecting each other much (*P* = 0.028 and 6.1e-10, respectively; model 2). Along with chronological age, however, AgeDiff was not significant at all, whereas FI_34_ maintained its significance (models 3–5). The Cox regression analysis was also repeated with AgeResid (Table 4), but AgeResid was not significant under any of the covariate combinations. As before, FI_34_ remained significant in all the models considered in Table 4.

**Table 3:** Cox regression for time-to-death as a function of AgeDiff or FI_34_ (*N* = 262)

Model	Variables	*b*	exp(*b*)	se(*b*)	*Z*	*P*	*R* ^2^	Wald test *P*
1	AgeDiff^c^	−0.017	0.98	0.0074	−2.3	0.020	0.022	0.053
2	AgeDiff^c^	−0.017	0.98	0.0075	−2.2	0.028	0.15	1.7e-09
	FI_34_^a^	0.049	1.05	0.0079	6.2	6.1e-10		
3	Age	0.12	1.13	0.011	11	<2.0e-16	0.48	<2.0e-16
	AgeDiff^c^	0.0037	1.00	0.0077	0.49	0.63		
4	Age	0.11	1.12	0.011	10	<2.0e-16	0.49	<2.0e-16
	AgeDiff^c^	0.0040	1.00	0.0078	0.52	0.61		
	FI_34_^a^	0.022	1.02	0.0080	2.8	0.0053		
5	Age	0.11	1.12	0.012	10	<2.0e-16	0.51	<2.0e-16
	AgeDiff^c^	0.0039	1.00	0.0084	0.47	0.64		
	FI_34_^a^	0.020	1.02	0.0085	2.4	0.016		
	WBC^b^	–	–		–	–		

All the regressions above contained sex as a covariate

*b* regression coefficient, *exp(b)* exponentiated *b*, *se(b)* standard error of *b*, *Z* the ratio of *b* to its standard error

^a^FI_34_ was multiplied by 100; therefore, the *b* value for FI_34_ is the hazard of death for an increase of FI_34_ by 0.01

^b^CD8.naive + CD8pCD28nCD45Ran + PlasmaBlast + CD4T + NK + Mono + Gran

^c^AgeDiff = AgeAccelerationDiff = DNAmAge − chronological age

**Table 4:** Cox regression for time-to-death as a function of AgeResid or FI_34_ (*N* = 262)

Model	Variables	*b*	exp(*b*)	se(*b*)	*Z*	*P*	*R* ^2^	Wald test *P*
1	AgeResid^c^	0.0023	1.00	0.0079	0.29	0.77	0.002	0.74
2	AgeResid^c^	0.00062	1.00	0.0081	0.076	0.94	0.13	1.3e-08
	FI_34_^a^	0.049	1.05	0.0079	6.2	5.2e-10		
3	Age	0.12	1.13	0.011	11	<2.0e-16	0.48	<2.0e-16
	AgeResid^c^	0.0037	1.00	0.0077	0.49	0.63		
4	Age	0.11	1.12	0.011	10	<2.0e-16	0.49	<2.0e-16
	AgeResid^c^	0.0040	1.00	0.0078	0.52	0.61		
	FI_34_^a^	0.022	1.02	0.0080	2.8	0.0053		
5	Age	0.11	1.12	0.012	9.8	<2.0e-16	0.51	<2.0e-16
	AgeResid^c^	0.0039	1.00	0.0084	0.47	0.64		
	FI_34_^a^	0.020	1.02	0.0085	2.4	0.016		
	WBC^b^	–	–		–	–		

All the regressions above contained sex as a covariate

*b* regression coefficient, *exp(b)* exponentiated *b*, *se(b)* standard error of *b*, *Z* the ratio of *b* to its standard error

^a^FI_34_ was multiplied by 100; therefore, the *b* value for FI_34_ is the hazard of death for an increase of FI_34_ by 0.01

^b^CD8.naive + CD8pCD28nCD45Ran + PlasmaBlast + CD4T + NK + Mono + Gran

^c^Residual = *y* − *ŷ* in linear regression of *ŷ* (DNAmAge) on *y* (chronological age)

### FI_34_ outperforms chronological age and DNAmAge-related measures in predicting mortality of nonagenarians

**Fig. 3:**
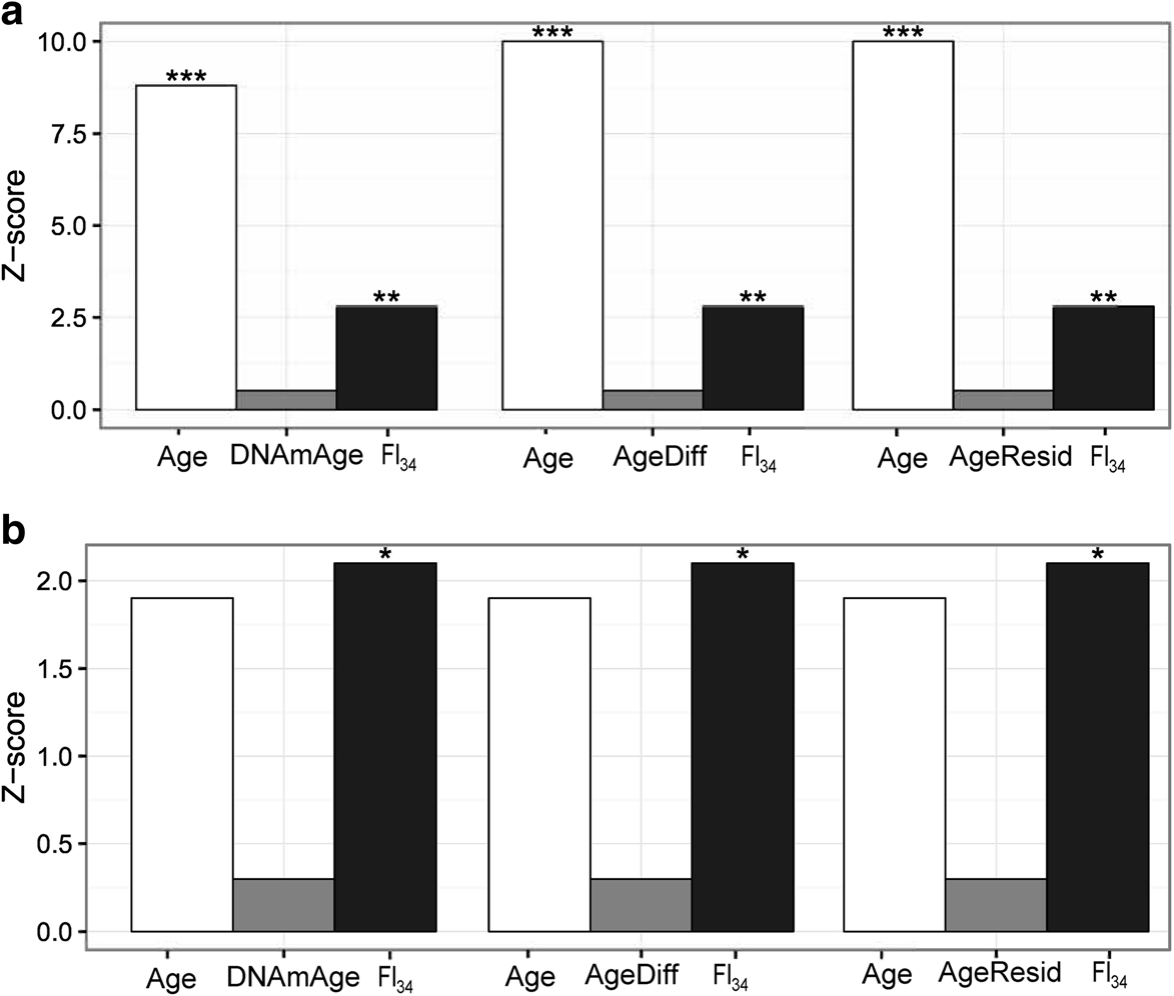
Bar plots of effect sizes (*Z* scores) from Cox proportional hazards regressions. **a** *Z* scores in model 7 of Table 2, model 4 of Table 3, and model 4 of Table 4 were plotted, with * representing 0.01 < *P* =< 0.05, ** 0.001 < *P* =< 0.01, and *P* =< 0.001. **b** Z scores from the same Cox regression models applied to nonagenarians only (*N* = 161)

In all the Cox regression models presented so far with the whole study cohort, which includes subjects of ages from 60 to 103, chronological age was the best predictor of mortality (Fig. 3a). When the Cox regression was limited to nonagenarians only, however, FI_34_ was a better predictor of mortality than chronological age (*P* = 0.035 vs. *P* = 0.054, respectively; Fig. 3b). This indicates that FI_34_ is a better measure of biological age at later years when accumulation of health deficits accelerates differentially among the oldest old.

## Discussion

Since Horvath’s calculation of DNAmAge based on genomic DNA methylation levels (beta values) and chronological age (Horvath 2013), two DNAmAge-derived measures have been used: AgeDiff and AgeResid. AgeDiff has been associated with mortality (Marioni et al. 2015; Christiansen et al. 2016; Perna et al. 2016) and a frailty index (Breitling et al. 2016), after adjustment for various sets of covariates, including the leukocyte type composition. More recently, Chen et al. expanded the original observation of Horvath using more than 13,000 individuals from 13 different cohorts, including three racial/ethnic groups, and found AgeResid to be a significant predictor of mortality (Horvath 2013; Chen et al. 2016). They also found that incorporation of leukocyte composition information greatly enhanced the significance of AgeResid in predicting mortality.

A true measure of biological age should predict mortality/survival with high accuracy. Our study showed that FI_34_ is a significant predictor of mortality, whereas DNAmAge, AgeDiff, and AgeResid are not, regardless of adjustment for leukocyte type composition. Our study sample consists of 262 Caucasians whose ages range from 60 to 103, and by comparing FI_34_ and each of the DNAmAge measures side by side, we clearly showed that FI_34_ is a far better predictor of mortality than the DNAmAge measures. DNAmAge was significant when the Cox regression was unadjusted for chronological age, but DNAmAge became nonsignificant when the regression was adjusted for this variable. This confirms a high degree of redundancy between DNAmAge and chronological age. The same is true with AgeDiff, the difference between DNAmAge and chronological age. Because DNAmAge and age are highly correlated with each other, AgeDiff is also correlated with age, though the correlation is not as strong as that of DNAmAge with chronological age. The negative correlation of AgeDiff with chronological age (Fig. 1c) indicates that the difference between DNAmAge and chronological age decreases as chronological age increases, which is due to decreasing DNAmAge with increasing chronological age. A similar observation was made in a longitudinal twin study (Christiansen et al. 2016). The cause of this leveling of DNAmAge relative to chronological age in later years of life remains to be determined. On the other hand, AgeResid, the residual of the linear regression of DNAmAge on chronological age, is not correlated with chronological age or DNAmAge. This is not surprising because residuals from linear regressions are not correlated with either of the variables used to calculate them. However, AgeResid was not a significant predictor of mortality in all the models we examined in our study. A larger sample size would likely give better linear regression fitting, which would yield better residual estimates. This could be one reason why our study could not detect a significance association of AgeResid with survival.

Our study used a cohort consisting of 262 subjects of European origin, whereas all the studies of DNAmAge used meta-analyses of the results from multiple cohorts involving many more subjects. A meta-analysis of combinable studies, if based on accurate statistical procedures without any bias, should give a higher statistical power than individual studies. Thus, it is possible that the meta-analysis studies involving large numbers of subjects were able to detect the significance of the various DNAmAge measures that our study was unable to. However, this consideration points to the fact that FI_34_ is a much more robust predictor of survival and measure of biological age than any of the DNAmAge measures proposed thus far. This is because it assesses biological factors that have large effects on survival, whereas the DNAmAge measures only detect small statistical differences which require very large samples. It is worth noting that this usually involves the use of leukocyte type estimates, which perhaps introduce some biological meaning that is sufficient to make them perform. FI_34_ is a particularly strong measure of biological age because it is a better predictor of survival than is chronological age (Fig. 3) in the oldest old.

## References

[CR1] Accomando WP, Wiencke JK, Houseman EA, Nelson HH, Kelsey KT (2014). Quantitative reconstruction of leukocyte subsets using DNA methylation. Genome Biol.

[CR2] Assenov Y, Muller F, Lutsik P, Walter J, Lengauer T, Bock C (2014). Comprehensive analysis of DNA methylation data with RnBeads. Nat Methods.

[CR3] Bocklandt S, Lin W, Sehl ME, Sanchez FJ, Sinsheimer JS, Horvath S, Vilain E (2011). Epigenetic predictor of age. PLoS One.

[CR4] Breitling LP, Saum KU, Perna L, Schottker B, Holleczek B, Brenner H (2016). Frailty is associated with the epigenetic clock but not with telomere length in a German cohort. Clin Epigenetics.

[CR5] Chen BH (2016). DNA methylation-based measures of biological age: meta-analysis predicting time to death. Aging.

[CR6] Christiansen L, Lenart A, Tan Q, Vaupel JW, Aviv A, McGue M, Christensen K (2016). DNA methylation age is associated with mortality in a longitudinal Danish twin study. Aging Cell.

[CR7] Fraga MF (2005). Epigenetic differences arise during the lifetime of monozygotic twins. Proc Natl Acad Sci U S A.

[CR8] Fried LP (2001). Frailty in older adults: evidence for a phenotype. J Gerontol A Biol Sci Med Sci.

[CR9] Hannum G (2013). Genome-wide methylation profiles reveal quantitative views of human aging rates. Mol Cell.

[CR10] Horvath S (2013). DNA methylation age of human tissues and cell types. Genome Biol.

[CR11] Horvath S (2015). Decreased epigenetic age of PBMCs from Italian semi-supercentenarians and their offspring. Aging.

[CR12] Houseman EA (2012). DNA methylation arrays as surrogate measures of cell mixture distribution. BMC bioinformatics.

[CR13] Houseman EA, Molitor J, Marsit CJ (2014). Reference-free cell mixture adjustments in analysis of DNA methylation data. Bioinformatics.

[CR14] Jazwinski SM (2010). HRAS1 and LASS1 with APOE are associated with human longevity and healthy aging. Aging Cell.

[CR15] Karasik D, Demissie S, Cupples LA, Kiel DP (2005). Disentangling the genetic determinants of human aging: biological age as an alternative to the use of survival measures. J Gerontol A Biol Sci Med Sci.

[CR16] Kim S, Jazwinski SM (2015) Quantitative measures of healthy aging and biological age. Healthy Aging Res 4:26. doi:10.12715/har.2015.4.26 10.12715/har.2015.4.26 PMC4440677 26005669

[CR17] Kim S, Myers L, Ravussin E, Cherry KE, Jazwinski SM (2016). Single nucleotide polymorphisms linked to mitochondrial uncoupling protein genes UCP2 and UCP3 affect mitochondrial metabolism and healthy aging in female nonagenarians. Biogerontology.

[CR18] Kim S, Simon E, Myers L, Hamm LL, Jazwinski SM (2016). Programmed cell death genes are linked to elevated creatine kinase levels in unhealthy male nonagenarians. Gerontology.

[CR19] Kim S, Welsh DA, Cherry KE, Myers L, Jazwinski SM (2013). Association of healthy aging with parental longevity. Age.

[CR20] Kim S, Welsh DA, Myers L, Cherry KE, Wyckoff J, Jazwinski SM (2015). Non-coding genomic regions possessing enhancer and silencer potential are associated with healthy aging and exceptional survival. Oncotarget.

[CR21] Kim S, Welsh DA, Ravussin E, Welsch MA, Cherry KE, Myers L, Jazwinski SM (2014). An elevation of resting metabolic rate with declining health in nonagenarians may be associated with decreased muscle mass and function in women and men, respectively. J Gerontol A Biol Sci Med Sci.

[CR22] Koch CM, Wagner W (2011). Epigenetic-aging-signature to determine age in different tissues. Aging.

[CR23] Kulminski A, Ukraintseva SV, Akushevich I, Arbeev KG, Land K, Yashin AI (2007). Accelerated accumulation of health deficits as a characteristic of aging. Exp Gerontol.

[CR24] Kulminski AM, Ukraintseva SV, Akushevich IV, Arbeev KG, Yashin AI (2007). Cumulative index of health deficiencies as a characteristic of long life. J Am Geriatr Soc.

[CR25] Lin Q, Weidner CI, Costa IG, Marioni RE, Ferreira MR, Deary IJ, Wagner W (2016). DNA methylation levels at individual age-associated CpG sites can be indicative for life expectancy. Aging.

[CR26] Marioni RE (2015). DNA methylation age of blood predicts all-cause mortality in later life. Genome Biol.

[CR27] Matteini AM (2010). Heritability estimates of endophenotypes of long and health life: the Long Life Family Study. J Gerontol A Biol Sci Med Sci.

[CR28] Mitnitski A, Bao L, Rockwood K (2006). Going from bad to worse: a stochastic model of transitions in deficit accumulation, in relation to mortality. Mech Ageing Dev.

[CR29] Mitnitski AB, Graham JE, Mogilner AJ, Rockwood K (2002). Frailty, fitness and late-life mortality in relation to chronological and biological age. BMC Geriatr.

[CR30] Mitnitski AB, Mogilner AJ, MacKnight C, Rockwood K (2002). The mortality rate as a function of accumulated deficits in a frailty index. Mech Ageing Dev.

[CR31] Mitnitski AB, Mogilner AJ, Rockwood K (2001). Accumulation of deficits as a proxy measure of aging. ScientificWorldJournal.

[CR32] Perna L, Zhang Y, Mons U, Holleczek B, Saum KU, Brenner H (2016). Epigenetic age acceleration predicts cancer, cardiovascular, and all-cause mortality in a German case cohort. Clin Epigenetics.

[CR33] R_Core_Team (2016). R: a language and environment for statistical computing.

[CR34] Rakyan VK (2010). Human aging-associated DNA hypermethylation occurs preferentially at bivalent chromatin domains. Genome Res.

[CR35] Reinius LE (2012). Differential DNA methylation in purified human blood cells: implications for cell lineage and studies on disease susceptibility. PLoS One.

[CR36] Rockwood K, Andrew M, Mitnitski A (2007). A comparison of two approaches to measuring frailty in elderly people. J Gerontol A Biol Sci Med Sci.

[CR37] Rockwood K, Fox RA, Stolee P, Robertson D, Beattie BL (1994). Frailty in elderly people: an evolving concept. CMAJ.

[CR38] Rockwood K, Mitnitski A (2007). Frailty in relation to the accumulation of deficits. J Gerontol A Biol Sci Med Sci.

[CR39] Rockwood K, Stadnyk K, MacKnight C, McDowell I, Hebert R, Hogan DB (1999). A brief clinical instrument to classify frailty in elderly people. Lancet.

[CR40] Searle SD, Mitnitski A, Gahbauer EA, Gill TM, Rockwood K (2008). A standard procedure for creating a frailty index. BMC Geriatr.

